# “I do not hear you!”: hearing-impaired cancer patients report their communication experiences

**DOI:** 10.1007/s00432-023-04634-0

**Published:** 2023-02-13

**Authors:** Maximilian Keck, Jutta Hübner, Jens Büntzel

**Affiliations:** 1 Department of Hematology and Medical Oncology, University Hospital Jena, Am Klinikum 1, 07747 Jena, Germany; 2 Department of Otolaryngology, Südharz Klinikum Nordhausen, Dr.-Robert-Koch-Str. 39, 99734 Nordhausen, Germany

**Keywords:** Cancer patients, Hearing impairment, Patient satisfaction, Physician–patient communication, APHAB, Hearing aids

## Abstract

**Purpose:**

Hearing impairment has a high impact on communication between cancer patients and their oncologists. What is the patient’s perspective on this problem and how can physicians draw lessons from it?

**Methods:**

Together with otorhinolaryngologists and hearing-impaired patients, we developed a questionnaire including the sections: WHO (Five) Well-Being Index (5 items), Abbreviated Profile of Hearing Aid Benefit (APHAB) as established self-estimation of hearing function (24 items), use of hearing aid (4 items) as well as patients’ experiences (15 items), difficulties (9 items) and wishes (7 items) regarding the communication with physicians. The experiences, difficulties, wishes, and life satisfaction of cancer patients were analyzed between groups based on participants' APHAB scores, well-being and use of hearing aids. A total of 104 cancer survivors (median age 76.5 years, range 32–90 years) were included.

**Results:**

Between the groups of subjectively hearing-impaired and normal hearing participants, we registered a significant difference in difficulties in conversation, wishes for physician–patient communication and psychological well-being. Depending on participants’ well-being, wishes and difficulties differed. Differences were also found between participants with and without hearing aids in terms of difficulties in conversation, but not in terms of their wishes and well-being. A large proportion of participants classified as subjectively hearing-impaired according to APHAB already had a hearing aid.

**Conclusions:**

Cancer patients with hearing loss are very restricted in their understanding of given information and hearing aid use cannot completely compensate for this. Consequently, communication guidelines should be considered and specific educational tools need to be developed for these groups.

## Introduction

As the world is in the midst of a major demographic change, physicians get used to have more and more elderly patients. Difficulties due to sensory impairments in this patient group may contribute to an inappropriate medical care. In particular, an increase of age-related hearing loss is expected in the next years because of the demographic transition (Ruf et al. 2016). In Germany, the prevalence of hearing impairment according to the WHO criterion is 16.2% in adults. This figure is expected to increase by around 1% per 5-year period (von Gablenz et al. 2017). At the same time, it is known that elderly people with hearing loss report poorer health and have a higher burden of disease (McKee et al. 2018).

Furthermore, patients with hearing loss have different preferences and needs for communication compared to normal hearing persons. Even if their auditory input is decreased, hard-of-hearing people heavily rely on speech and audition. Thus, they are still able to derive linguistically useful information from dialogs (Münstermann et al. 2022).

To provide effective and efficient services to these individuals, it is important to comprehend the effects of hearing loss on satisfaction with medical care (Barnett et al. 2014).

Particularly when it comes to patient–physician communication in the context of serious and life-limiting illnesses, sufficient understanding is crucial and might affect outcomes of treatment protocols as well as the patient’s life quality. This is why the Institute of Medicine (Schickedanz 2009) emphasized the need to improve communication with patients suffering from cancer. To achieve progress in this regard, it is essential for medical providers to be well-informed about the patients’ hearing abilities.

Until now, hearing loss is often undetected (Li-Korotky 2012). Thus both the physician and the patient may not be aware of it. Especially, presbycusis is characterized by a creeping onset giving patients the chance to develop more or less effective compensation strategies in daily life activities. That’s why hearing difficulties are oftentimes negated at first, subsequently repressed (Löhler et al. 2013) and, therefore, not revealed to the physician. The impact could be significant if assignments, clarifications or advice are given to the patient (Zeitlin 2016). As a consequence, cancer patients might miss important information with negative consequences on the course of their disease and satisfaction with the received care.

In fact, compared to people with unrestricted hearing, deaf and hard-of-hearing persons have less knowledge about recommended prevention interventions against cancer (Zazove et al. 2009). Moreover, their attitudes toward these interventions are influenced by their own experiences with physicians (Tamaskar et al. 2000). Our study seeks to find out whether psychological well-being and experiences in physician–patient communication with resulting problems and wishes may vary depending on the hearing ability of patients. To improve the satisfaction of hearing-impaired cancer patients, it is necessary to ask them about concrete difficulties and wishes in typical situations in medical practices. The purpose of this study is to provide an overview of how their experiences differ from normal hearing people. With these findings, it may be possible to provide new ideas and better medical communication with hearing-impaired people suffering from cancer.

In particular, this study assesses differences in well-being, problems and wishes in conversations between (1) cancer patients with and without subjective hearing loss, (2) cancer patients wearing a hearing aid and those without and (3) cancer patients with poor well-being and those with good well-being. Furthermore, the use and the satisfaction with hearing aids of cancer patients using them and specific experiences in conversations during medical consultation will be evaluated. The findings may contribute to a better detection of hearing loss among individuals with cancer and to identify proper ways of how to effectively communicate and share important medical information with them.

## Methods

### Participants

Cancer patients were identified based on their known medical history and were invited to participate in the offices of an ophthalmologist and an otorhinolaryngologist in the city of Weissenfels (Saxony-Anhalt, Germany). Participation was anonymous and voluntary. Informed consent was obtained from all individual participants included in the study. Between 01-04-2021 and 31-07-2021, 104 attendees filled out the questionnaire.

### Questionnaire

The questionnaire contained eight main sections:

1. General information on the study (institution, aims, informed consent).
2. Demographic data: Gender, age, education level, profession, cancer anamnesis and localization of cancer.
3. WHO (Five) Well-Being Index (WHO-5): With its high clinimetric validity, this questionnaire is one of the most commonly used questionnaires to assess subjective psychological well-being (Topp et al. 2015). It contains five items which can be answered on a Likert scale ranging from “5—All the time” to “1—At no time.” Poor well-being and indication to screen for depression according to ICD-10 is indicated by a score below 13 (WHO 1998). Since hearing loss appears to be associated with a statistically significant higher probability of depression in older adults (Lawrence et al. 2020), it is important to take a look at the well-being of our patients as it may influence outcome.
4. Abbreviated Profile of Hearing Aid Benefit (APHAB): This questionnaire is established for self-estimation of hearing function containing 24 items in four sections: ease of communication, background noise, reverberation and aversiveness of sounds. The scaled answers are allocated to percentages. A correctly answered questionnaire to form a total APHAB score contains of at least 4 out of 6 answered questions of each category (Löhler et al. 2017). The values of this questionnaire are independent from hearing loss diagnosed with pure-tone audiograms, but provide information about the subjective hearing ability of patients (Löhler et al. 2016).
5. Usage of hearing aid: Use of hearing aid (none or the type of hearing aid), regularity of use, satisfaction, evaluation of hearing enhancement.
6. Experiences in direct conversations with physicians: Participants should evaluate typical situations in conversations with their physicians on a Likert scale ranging from “1—not applicable” to “4—fully applicable” and “0—not sure.” The 15 items were taken from a questionnaire by Münstermann et al. (2022).
7. Difficulties in conversation: Nine items to answer with the similar Likert scale as described above. To ensure the content validity, the questions were developed with the help of two otorhinolaryngologists.
8. Further wishes regarding the communication with physician: Seven items with similar Likert scale. To ensure the content validity, the questions were developed with the help of two otorhinolaryngologists.

### Statistics

All data were collected in MS Excel. For statistical analysis, the free software GNU PSPP was used.

### Forming new variables

For reliability analysis, Cronbach’s alpha was calculated to assess the internal consistency of the subscale “It´s hard for me to…” (seventh section) and “I would wish, that…” (eighth section). The internal consistency of the scale “It´s hard for me to…” which consists of nine items is satisfying, with a Cronbach´s alpha for positive effect = 0.89. A comparable positive effect was proven for the scale “I would wish, that…” with a Cronbach´s alpha of = 0.82. Consequently, two new variables could be generated which reflect the difficulties and wishes in direct conversations with their physicians.

### Group analysis

One of our aims was to see whether a tendency for subjective hearing loss or wearing a hearing aid has an impact on difficulties in talking with their physician, wishes for physician–patient communication and well-being. For this purpose, all participants were first divided into groups according to their APHAB scores (APHAB < 40%: no tendency for subjective hearing loss; APHAB $\ge$ 40%: tendency for subjective hearing loss). Second, participants were divided into groups according to whether or not they had a hearing aid. The cut-off in the APHAB questionnaire was defined upfront. Therefore, some results of speech audiometry performed in one of the participating offices were compared with the APHAB scores of the same patients. The cut-off value of 40 points was determined together with two otorhinolaryngologists who use the APHAB questionnaire constantly in their practices. Another aim was to figure out if the overall well-being impacts difficulties in and their wishes or expectations for conversations with their physicians. In this regard, we split all data into groups of participants with WHO scores over and under 13 points. To analyze the differences in experiences in direct conversation with physicians, the responses from hearing-impaired and normal hearing participants were compared. Beside frequency analysis and descriptive statistics, significances were tested by the non-parametric Wilcoxon-Mann–Whitney test for independent samples. To determine the effect size of the Wilcoxon-Mann–Whitney tests performed, the Pearson correlation coefficient *r* was calculated and interpreted according to Cohen (1988). A *p* value below 0.05 was considered significant.

In the following, we will refer to participants identified as subjectively hard of hearing (which is consistent with the validity of the APHAB questionnaire) as hearing-impaired or hard-of-hearing participants.

## Results

### Demographic data

One hundred and four participants completed the questionnaire. Age ranged between 32 and 90 years, with a mean of 73.4 years and median of 76.5 years. Forty-nine (47.1%) of the interviewed participants were male, fifty-five (52.9%) were female. The largest group (84.6%) were pensioners; more than half of the participants (66/102) reported high education levels (secondary school qualification, university entrance diploma, university degree).

Detailed demographics of participants in the survey are shown in Table 1.

**Table 1:** Demographic data of participants (*N* = 104)

	N (% of valid answers)
Gender	*N* = 104
Female	55 (52.9)
Male	49 (47.1)
Age	*N* = 102
< 40	1 (1.0)
41–60	9 (8.8)
61–70	26 (25.5)
> 70	66 (64.7)
Education level	*N* = 102
No qualification	2 (2.0)
Lower secondary school leaving certificate	34 (33.3)
Secondary school qualification	31 (30.4)
University entrance diploma	11 (10.8)
University degree	24 (23.5)
Occupation	*N* = 104
Working	14 (13.5)
Pensioner	88 (84.6)
Jobless	2 (1.9)
Cancer anamnesis	*N* = 104
Positive	104 (100)
Negative	0 (0)

### Well-being

Eighty-six of all participants answered the WHO (Five) Well-Being questionnaire. Of these, 62.8% (54/86) scored above 13, 32/86 participants (37.2%) showed scores below 13, which indicates poor well-being and potentially depression (WHO 1998).

### Hearing ability

Barely more than half of all participants (51/97, 52.6%) did not show values for subjective hearing impairment by reaching an APHAB score below 40%. The other forty-six participants (46/97, 47.4%) were registered as tending to subjective hearing difficulties.

### Hearing aid

**Fig. 1:**
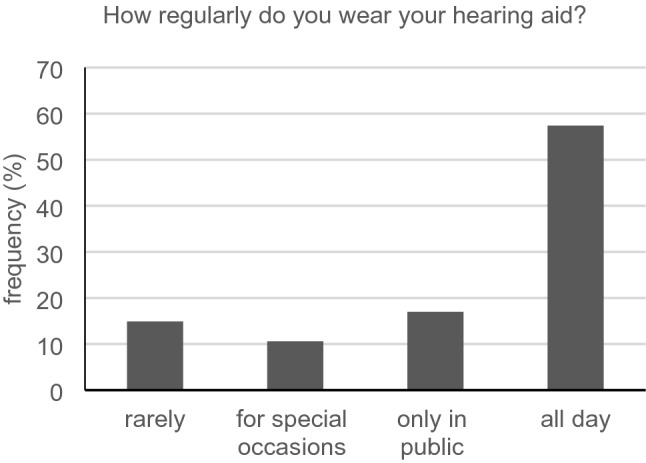
Regularity of hearing aid use (*n* = 47)

**Fig. 2:**
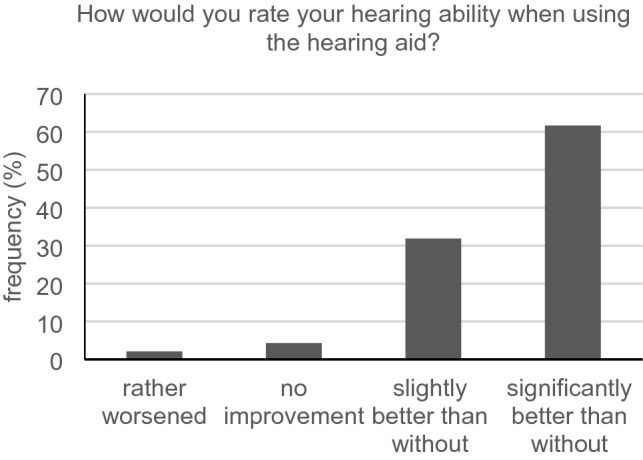
Hearing improvement (*n* = 47)

Forty-eight of all participants reported having a hearing aid. Only 57.4% (27/47) of them wore them all day long (Fig. 1). Most participants with a hearing aid reported benefit (44/47, 93.6%) (Fig. 2), 39/46 (84.8%) were either rather satisfied, satisfied or very satisfied. In the group of participants showing a subjective hearing loss (APHAB score of 40 or superior), we registered 29 people (29/44, 65.9%) wearing a hearing aid.

### Group comparison by hearing ability

As described, all participants who answered the APHAB questionnaire correctly were separated into two groups. We registered a significant difference between participants with and without hearing loss tendency in difficulties in the conversation with their physician (2.30 ± 0.67 vs. 1.66 ± 0.74, *U* = 459.50, *Z* = − 4.47, *p* < 0.001). According to Cohen (1988), the interpretation of the Pearson correlation coefficient “*r*” (effect size) results in a mean effect (*r* = − 0.471). Similar significant difference was identified in desires for physician–patient communication (1.97 ± 0.75 vs. 1.60 ± 0.68, *U* = 606.50, *Z* = − 2.48, *p* = 0.013).

Moreover, a significant difference was detected by having a look at people’s well-being (12.35 ± 5.66 vs. 15.77 ± 5.97, *U* = 525.00, *Z* = − 2.75, *p* = 0.006) with a mean effect according to the Pearson correlation coefficient (*r* = − 0.306).

### Group comparison by hearing aid use

Nearly half of all participants wore a hearing aid (48/102, 47.1%) and 54/102 did not (52.9%). A significant difference with a mean effect (*r* = − 0.361) could be shown in difficulties in conversation (2.28 ± 0.79 vs. 1.72 ± 0.65, *U* = 628.50, *Z* = − 3.48, *p* < 0.001). A significant difference could not be seen in either wishes for conversations with their physician (1.96 ± 0.79 vs. 1.64 ± 0.67, *U* = 688.00, *Z* = − 1.90, *p* = 0.057) or in level of well-being (14.61 ± 5.74 vs. 13.67 ± 6.27, *U* = 811.50, *Z* = − 0.56, *p* = 0.574).

### Group comparison by well-being

Between participants with WHO-5-scores below 13 and those above, a significant difference in difficulties in talking could be shown (2.19 ± 0.67 vs. 1.78 ± 0.66, *U* = 494.50, *Z* = − 2.62, *p* = 0.009). Further on, wishes for discussions between patient and physician were significantly different in groups of people with good and poorer well-being; (2.03 ± 0.78 vs. 1.58 ± 0.66, *U* = 383.00, *Z* = − 2.52, *p* = 0.012). Both correlations showed a low effect (*r* = − 0.293 and *r* = − 0.299) according to Cohen (1988).

### Experiences of hearing-impaired individuals in direct conversations with physicians

**Fig. 3:**
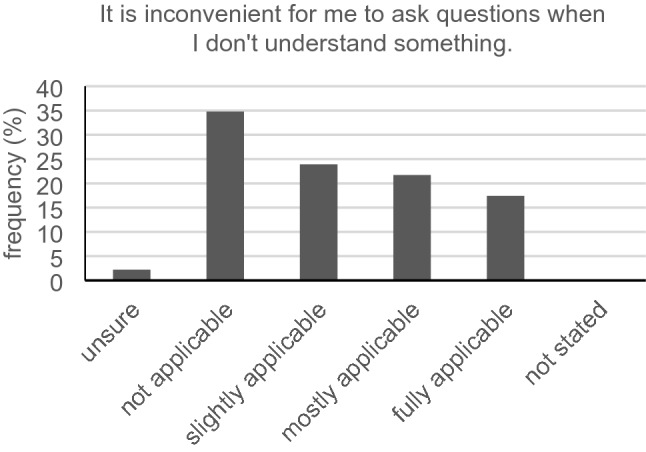
Inconvenience with queries (*n* = 46)

**Fig. 4:**
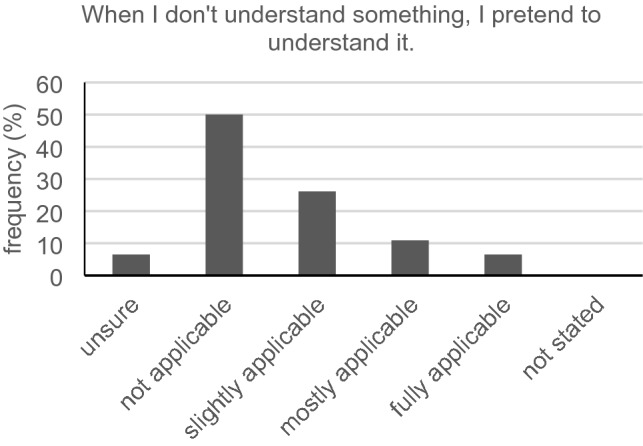
Feigning understanding (*n* = 46)

Only 7/46 participants (15.2%) with subjective hearing difficulties reported problems to announce themselves at the reception. A total of 17.4% (8/46) fully agreed with the statement that it is easy for them to understand the physician´s speech. The majority (24/45, 53.3%) must mostly or always concentrate very well to understand the words and phrases of the physician. According to the given answers, only 13 out of 46 participants (28.3%) understood all verbal instructions for behavior patterns, medication and medical examinations given by their physician. Furthermore, it was sometimes, mostly or always inconvenient for 63.0% (29/46) for the hearing-impaired asking back if they partly did not understand what had been said by the physician (Fig. 3). Just 28.9% (13/45) told their physician they were a hard of hearing person and asked them to speak loudly and clearly. The vast majority (39/46, 84.8%) requested a repetition by the physician in case they did not understand what had been said the first time. Exactly half of all participants (23/46, 50.0%) stated that they never pretend to understand something, even if they do not (Fig. 4). Five of our participants (5/45, 11.1%) felt like the physician does not take their hearing loss into consideration during the conversation. Less than half (21/45, 46.7%) thought that their physicians only focus on them during the conversation and are not distracted all the time. A total of 56.5% (26/46) reported the physician holds eye contact during conversation and 54.3% (25/46) declared the physician speaks loudly and clearly. Asking the participants if their physician deals well with the problems of understanding in case of misunderstandings, 57.8% (26/45) answered with “sometimes” and “mostly.” Only 10 out of 44 participants (22.7%) with hearing impairment said that the physician regularly asks if they can mentally follow him or her. For 86.7% (39/45), wearing a surgical face mask sometimes, mostly or always constituted a greater difficulty for understanding what is being said.

### Difference between normal hearing and hearing-impaired patients

**Fig. 5:**
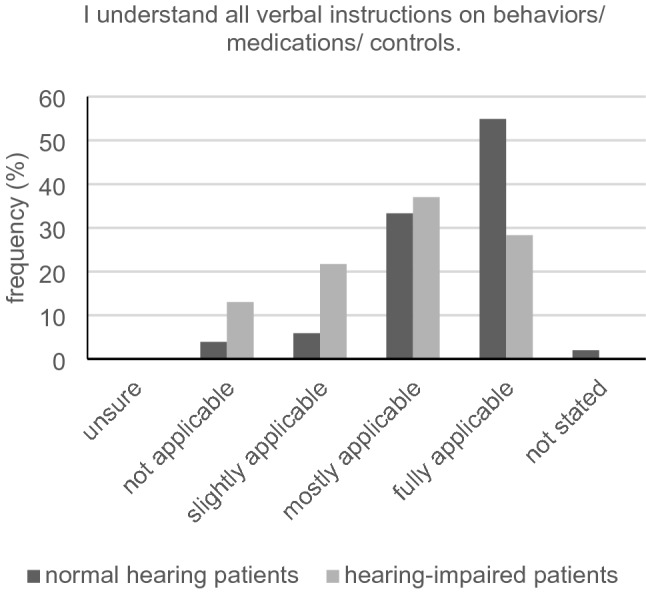
Understanding verbal instructions in the group of normal hearing (*n* = 51) and hearing-impaired (*n* = 46) patients

**Fig. 6:**
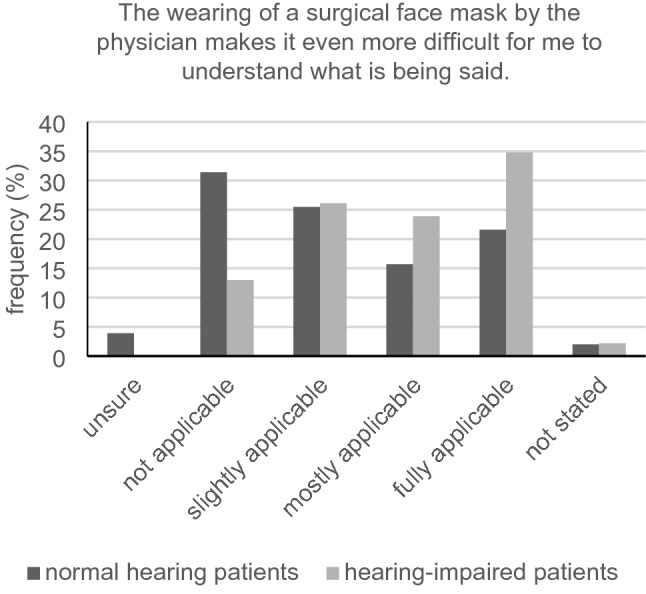
Effects of surgical face masks on comprehension in the group of normal hearing (*n* = 51) and hearing-impaired (*n* = 46) patients

With respect to the statement “I can easily understand the physician's sentences,” we registered a significant difference between participants with and without hearing loss (2.70 ± 0.99 vs. 3.44 ± 0.58, *U* = 647.00, *Z* = − 4.03, *p* < 0.001) with a strong effect (*r* = − 0.594). Furthermore, a significant difference could be measured when being asked if they have to concentrate a lot to hear the physician’s words and phrases (2.51 ± 1.18 vs. 1.92 ± 1.07, *U* = 787.00, *Z* = − 2.61, *p* = 0.009). Similar significant difference between the two groups was seen in the statement “I understand all verbal instructions on behaviors/ medications/ controls” (2.80 ± 1.00 vs. 3.42 ± 0.78, *U* = 738.50, *Z* = − 3.23, *p* = 0.001) with a mean effect (*r* = − 0.476) (Fig. 5). Questioning the participants whether they are uncomfortable asking if they have not understood something, a significant difference was seen between the group with and without tendency for hearing impairment (2.17 ± 1.16 vs. 1.43 ± 0.79, *U* = 700.50, *Z* = − 3.48, *p* < 0.001). No significant difference could be measured in relation to the statement “If I don't understand something, I always ask for it to be explained to me again” (2.83 ± 1.10 vs. 3.00 ± 1.03, *U* = 1051.50, *Z* = -0.76, *p* = 0.449). By asking if wearing a surgical face mask makes it even more difficult for participants to understand what is being said, a significant difference between the two groups was seen (2.82 ± 1.07 vs. 2.20 ± 1.23, *U* = 799.00, *Z* = − 2.51, *p* = 0.012) with a mean effect (Pearson correlation coefficient *r* = − 0.374) (Fig. 6).

## Difficulties in conversation in hard-of-hearing patients

**Fig. 7:**
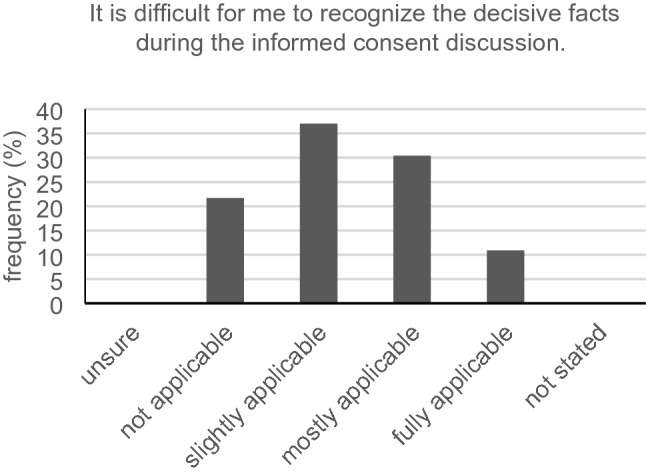
Recognizing decisive facts (*n* = 46)

**Fig. 8:**
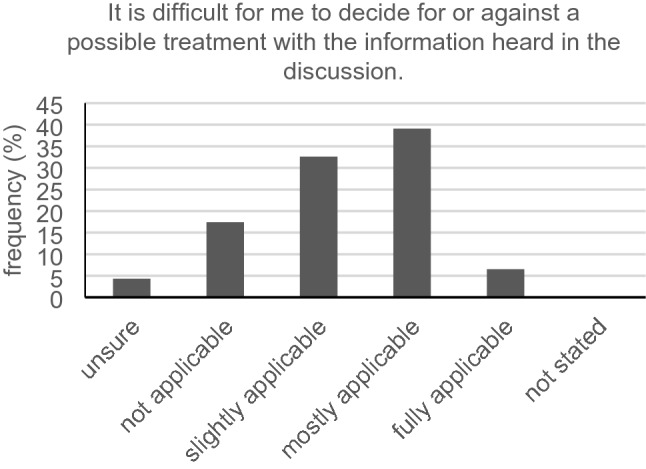
Decision-making based on the information heard (*n* = 46)

**Fig. 9:**
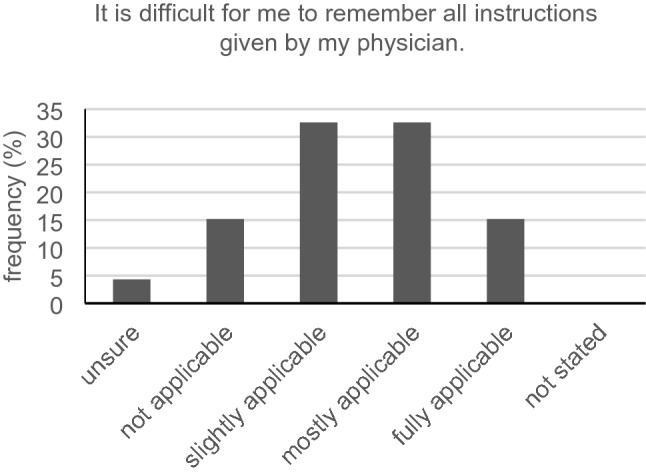
Remembering instructions (*n* = 46)

Only 16 out of 46 participants (34.8%) with hearing loss had no problem telling their physician they partly did not understand what had been said in the conversation. The vast majority (36/46, 78.3%) could slightly, mostly or fully relate to the statement: “It is difficult for me to recognize the decisive facts during the informed consent discussion” (Fig. 7). Keeping eye contact during conversation was a huge problem for 23.9% (11/46). Another major issue for the hearing-impaired was capturing all the information in conversation when the physician speaks a little faster than usual. Forty participants (40/46, 87.0%) could slightly, mostly or fully relate to this statement. Just 12 out of 45 participants (26.7%) did not have trouble answering queries in informed consent discussion. Exactly 39.1% (18/46) mostly agreed with the statement that it is hard for them to decide for or against a possible treatment with the information heard in the discussion (Fig. 8). The same average answer was given from 17/46 (37.0%) as to whether it is hard for them to understand the full scope of a treatment. Over 80% (37/46, 80.4%) of our hard-of-hearing participants slightly, mostly or always had trouble remembering all instructions given by their physician (Fig. 9). Only seven participants (7/46, 15.2%) had no problems interpreting the physician’s facial expression when wearing a surgical face mask.

## Wishes for the physician–patient communication among participants with hearing loss

**Fig. 10:**
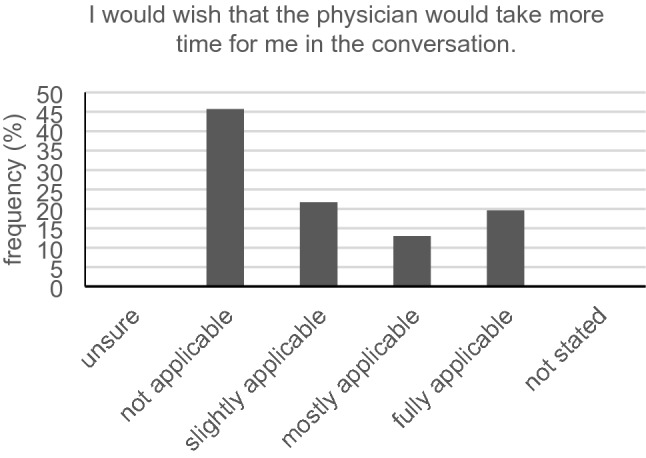
Time in conversation (*n* = 46)

**Fig. 11:**
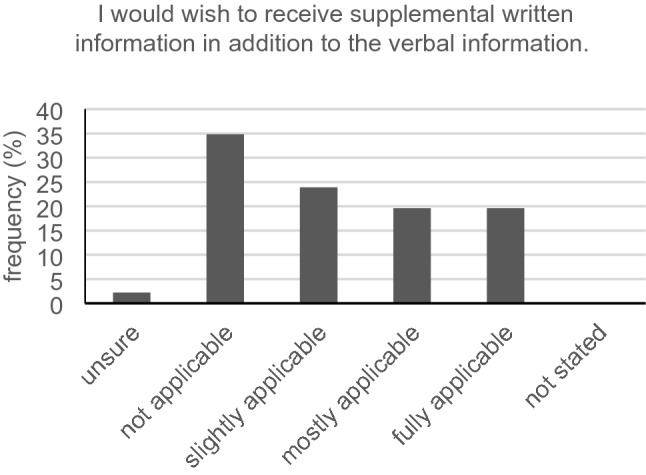
Receiving written information (*n* = 46)

Precisely half of all hearing-impaired participants (23/46, 50.0%) wished that the medical staff would take more time for them at the front desk. Furthermore, 54.3% (25/46) partly, mostly, or fully agreed with the statement: “I would wish that the physician would take more time for me in the conversation” (Fig. 10). Less than half (18/45, 40.0%) did not want the physician asking them multiple times if they understood what had been said. Asking our hard-of-hearing cancer participants if they would like to receive supplemental written information in addition to the verbal information, the majority (63.0%, 29/46) responded with “sometimes,” “mostly” or “always” (Fig. 11). The vast majority (38 of 44, 86.4%) did not need a sign language or speech-to-text interpreter. Eye contact during most of the time in the conversation was wished by a great majority of participants (35/46, 76.1%). A little more than half (54.3%, 25/46) did not want to receive all information and instructions previous to their talk with the physician.

## Discussion

To ensure good quality of instruction, education, and counseling, the high prevalence of hearing impairment in cancer patients must be considered. If not specifically asked for, they otherwise might have been overlooked in everyday practice.

### Depressive moods among hearing-impaired cancer patients

A large proportion of our participants suffer from reduced psychological well-being. For these individuals, the indication for screening for depression would be given (WHO 1998). The long-known fact that older adults with hearing loss may be at increased risk for depression (Lawrence et al. 2020) has been substantiated. Consequently, appropriate screening for this appears to be useful. Furthermore, our results coincide with previous findings of Massie (2004) that cancer at any site and stage of the disease is associated with high degrees of depression. The prevalence in this group of individuals is ranging from 0 to 58% (Massie 2004). In summary, the possibility of depressed mood in hearing-impaired people should be kept in mind.

### Hearing aid benefits

Despite satisfaction with the hearing aid, the proportion of those who reported wearing it all day seems unsatisfactory. This finding matches with the survey of Bainbridge and Ramachandran (2014). In this study, epidemiological data from 2005 to 2006 were compared with data from 2009 to 2010. A representative sample of 1,636 hearing-impaired people over the age of 70 years was surveyed regarding their treatment with a hearing aid and actual use. Only one-third of the hearing aid candidates in this study wore the device for at least five hours per week. These findings show the continuing poor acceptance of hearing aids, especially among older people. Since similar results are emerging in our study, it seems important for physicians to ask their patients if they have hearing aids. If so, they should wear them during the physician–patient communication.

Based on our findings, wearing a hearing aid does not guarantee fully restored hearing. Hearing aid users probably cannot act like normal hearing people in physician–patient conversations. For this reason, they should still be considered as a separate patient group. This seems to match with the results of Stengel et al. (2014) who, in an APHAB-based survey of more than 6000 hearing aid treated patients in Germany, found that only 70% really profited from their aid. However, it should be mentioned that the benefit again depends on the regular wearing of the hearing aid.

In our study, hearing aid users have more difficulties in physician–patient communication than those without hearing aids. This could mean that potential conversational difficulties are not automatically eliminated by simply wearing a hearing aid. This further reinforces our recommendation that physicians should continue to consider this group as handicapped compared to people with normal hearing. Patients with and without hearing aids appear to have similar wishes for physician–patient interaction. This may indicate that wearing a hearing aid improves communication and reduces desires regarding conversation. Also, the well-being of these two groups seems to be similar. This coincides with the finding of Mondelli and de Souza (2012) who found a significant improvement in quality of life after hearing aid fittings. Accordingly, a hearing aid supply makes sense and puts patients in a better position when it comes to comprehension of important instructions or advice.

### Influence of hearing loss on the physician–patient communication

In our study, it could be found that hearing-impaired cancer participants had more problems and wishes when talking to their physicians compared to normal hearing participants. Even their well-being was worse. This again emphasizes the relevance of our study and thus the importance of focusing on cancer patients who are hearing-impaired.

#### Experiences in conversation

It seems that it is more difficult for the hearing-impaired to understand what the physician has said and that this involves a greater effort of concentration for them. Increased concentration while listening is one of the first steps in the development of unconscious compensatory strategies. This permanently increased concentration subsequently leads to increased listening effort. This may result in a reduced attention span and a premature concentration deficit in conversation (Zorn and Lenker 2018).

Willingness to ask the physician for explanation seems to be similar to that of normal hearing individuals. Nevertheless, queries appear to be more unpleasant for the hearing-impaired. This is in line with a well-known behavior in this group of people. Some people withdraw from the conversation and try to give the impression they have understood everything. The other possibility to cover up their hearing loss would be to dominate the conversation partners and to determine the course of conversation (Zorn and Lenker 2018). This strategy might be difficult to implement in the physician–patient communication. Maybe that is why patients tend to move into a defensive position. Additionally, hearing-impaired individuals sometimes feel “guilty” for the extra effort it takes for the physician to communicate with them (Barnett 2002). Feigning to be able to follow the conversation seems to be a strategy of hard-of-hearing cancer patients. Concluding from this, regular check-backs may be a good option to avoid unpleasant queries for the patient.

Even if they are aware of their hearing loss, hearing-impaired patients are very unlikely to mention their impairment during physician–patient communication. Thus, it remains the physician's task to recognize a possible hearing loss. It is necessary to give patients the feeling that they are the center of attention. For example, it is important not to appear distracted, to maintain eye contact as consistently as possible and to speak clearly and distinctly. These simple prerequisites should be considered more often by physicians. In times of having to wear masks due to the Corona pandemic, these items are even more important.

#### Difficulties in conversation

For a big number of our hearing-impaired cancer patients, it caused troubles to recognize the decisive facts during informed consent discussion. This leads to the claim that it could be necessary to develop further formats which are adapted to the needs of hearing-impaired patients who rely on auditory input (Münstermann et al. 2022). Furthermore, it seems difficult for hearing-impaired patients to grasp all the information in a conversation when the physician is talking a little faster than usual. This problem needs to be solved but is restricted by the time limits in physicians’ offices. The consequence of rushing through informed consent discussions is that many patients seem overwhelmed by the flood of information. This makes it difficult for them to remember all of the instructions, to understand the full scope of a treatment and to decide for or against a possible treatment based on the information heard in the discussion.

Covering the physicians’ facial expressions and movement of the mouth, surgical face masks lead to comprehension problems for our hearing-impaired participants. Furthermore, face masks muffle sound (Mheidly et al. 2020) and make communication even more difficult for the hearing-impaired. In a survey by Poon and Jenstad (2022), over 80% of 656 self-identified deaf or hard-of-hearing adults reported troubles with understanding people wearing a face mask. As a result, the authors proposed several recommendations to improve communication. Among other things, they suggested the use of clear face masks to assist with lip reading. They also recommended more education on how to communicate more effectively with hearing-impaired adults while wearing a face mask.

#### Desires for conversation

Irving et al. (2017) systematically reviewed articles reporting on the duration of physician–patient consultations in primary care. In 18 countries representing approximately 50% of the world's population, mean duration was 5 min or less. Germany is only slightly above average with a mean duration of 7.6 min. In line with this, half of the hearing-impaired participants wished to have more time with the physician and the medical staff at the front desk. Meeting the special needs of a hearing-impaired patient in such a short time is a challenge. Regular queries from the physician during the conversation are useful to ensure that the patient follows what is being said. However, our study shows that not all hearing-impaired cancer patients want these questions repeated multiple times in a consultation. Additional information was welcomed by the majority but not previous to their talk with the physician. Eye contact seems to be wished and is important for easy grasping of information (Kee et al. 2018). Yet, due to the need for digital documentation, this is not always easy to realize.

### Consequences for communication in practice

Based on Barnett (2002) and our findings, we developed detailed recommendations for the physician–patient communication with hearing-impaired individuals, summarized in Table 2.

**Table 2:** Communication guidelines with hearing-impaired patients

*Before conversation*
Ask the patient if they have any specific requests for communication
Ask the patient if they have a hearing aid and if so, ask them to wear it
*Surrounding*
Provide a quiet environment
Do not place any objects between you and the patient that could cover your face
*During conversation*
Speak loud and clearly
Establish eye contact
Make the patient feel you have enough time for him/her
Ask for repetition by the patient but only in case of information that is important
If a patient does not understand, repeat the information or rephrase your statement if necessary
Provide your patient with a written summary of the particularly important information discussed after the consultation

## Limitations

In general, questions about satisfaction in the physician–patient communication vary by physician. The survey is limited by the fact that it was partly conducted in an otorhinolaryngology practice. For this reason, the proportion of hearing-impaired persons and hearing aid users was comparatively high. Moreover, the staff of an otorhinolaryngology office is well-trained in dealing with hard-of-hearing patients. For this reason, the data on difficulties and wishes for the interview could be somewhat distorted into the positive. It should be considered that some of the cancer patients related the survey to the practice they are in during the survey rather than judging their general experience with physician interviewing. Another factor influencing the results may also be the exceptionally high level of education of our participants. An important point to note is the fact already been explained in the beginning, in that this study refers to the subjective hearing loss of the participants as determined by the APHAB questionnaire. Thus, a profound diagnosis of hearing loss cannot be made. Despite this difference, the conclusions can be considered as general. Since we did not specifically ask for reasons, we cannot clarify why only about half of our hearing aid users wore their device all day and why not all users are satisfied with their hearing aid. The survey was conducted during the COVID-19 pandemic. However, no lockdown was prevalent at the time of survey execution. Bäuerle et al. (2021) described an increase of symptoms in major depression among tumor patients from 9.3 to 16.7% and an increase of symptoms in generalized anxiety disorder from 8.0 to 20.7% in March 2020. On the one hand, this circumstance could have influenced our examination of psychological well-being. On the other hand, such an increase in depressive moods may shift satisfaction with the physician’s conduct of the interview into the negative.

## Conclusion

The proportion of participants with subjective hearing difficulties among cancer patients was quite high. For this reason, it is important to always keep in mind the possibility that patients may not be able to follow the conversation adequately. In addition, the psychological well-being of patients with reduced hearing is significantly worse. The patient may, therefore, be preloaded with depressive traits. To identify depressive mood prior to the start of a conversation, the WHO (Five) Well-Being Index (WHO-5) is a proven tool and can be used in daily practice.

Hearing aid fitting itself does not guarantee normal hearing. Therefore, it may be necessary to continue to consider these individuals as handicapped in communication. However, if physicians get to know about a hearing impairment, they should actively suggest using a hearing aid in the conversation.

Physicians should not rely on patients reporting about their hearing loss on their own initiative at the beginning of the conversation. The same applies if patients are actually aware of their hearing loss. For at least roughly verifying a suspected hearing loss that has not been clarified, developing shorter questionnaires constitutes an alternative. An example is the Mini Audio Test (MAT) from Löhler et al. (2013). This short questionnaire consists of only six questions and is designed for a patient group aged 50–75 years. It can give a first, quite accurate indication of any hearing loss present.

Guidelines on how to communicate with hearing-impaired patients should be followed. Even with normal hearing individuals, physicians should pay attention to better articulation and slower focused speech.

Hearing-impaired patients were more uncomfortable asking for clarification if they did not comprehend facts. For this reason, it seems advisable to regularly inquire whether the patient has understood what has been said. In some cases, our participants seemed to have enormous problems in the physician–patient communication. Thus, the personal brief educational interview cannot always be a good and reliable basis for a self-determined decision for or against treatment. In consequence, further alternative offers should be created.

## Data Availability

The dataset generated and analyzed during the current study is available from the corresponding author on reasonable request.
